# Three-dimensional power Doppler ultrasound evaluation of placental blood flow in normal monochorionic diamniotic twin pregnancies

**DOI:** 10.1186/s12884-018-2080-y

**Published:** 2018-11-14

**Authors:** Wei Sun, Shaowei Yin, Qiuju Wei, Ying Zhang, Zeyu Yang, Ailu Cai, Yu Wang, Wenjia Lei

**Affiliations:** 1 Department of Sonography, Shengjing Hospital of China Medical University, Shenyang, China; 2 Department of Obstetrics and Gynecology, Shengjing Hospital of China Medical University, Shenyang, China; 3 Department of Obstetrics and Gynecology, the University of Tennessee Health Science Center, Memphis, TN USA

**Keywords:** Monochorionic, Three-dimensional power Doppler, Twin, Prenatal ultrasound, Placenta

## Abstract

**Background:**

Monochorionic diamniotic (MCDA) twin pregnancies are at higher risk of adverse outcomes and complications, which are attributed to the influence of placental morphology in MCDA twins. Monitoring of placental function is an important index for clinical decisions. The aim of our study was to evaluate the placental blood flow estimated using three-dimensional power Doppler (3D-PD) ultrasound and the vascular indices distribution with gestational age (GA) in normal MCDA twin pregnancies.

**Methods:**

One hundred four MCDA twin pregnancies and 106 singleton pregnancies (GA range, 14–32 weeks) were included in this prospective study. 3D-PD volume data of each fetus was obtained separately from the placenta at the site of umbilical cord insertion. We analyzed the volume data using sonobiopsy technique. The placental vascularization index (VI), flow index (FI) and vascularizationflow index (VFI), were auto-calculated. The means and standard deviation values of three vascular indices per fetus were calculated and regression analysis of the vascular indices as a function of GA was performed in twin pregnancies. The vascular indices of twin and singleton pregnancies were compared using independent *t*-test.

**Results:**

There were no significant differences in VI, FI or VFI among the fetuses of twins (*p* > 0.05). These vascular indices increased over the course of pregnancy (*p* < 0.05). We obtained the regression equations for the indices as a function of GA in days: VI = exp. (4.369–28.533/GA) (*R*^*2*^ = 0.699, *p* < 0.05), FI = exp. (3.916–13.003/GA) (*R*^*2*^ = 0.511, *p* < 0.05), and VFI = exp. (3.577–37.468/GA) (*R*^*2*^ = 0.675, *p* < 0.05). There were no significant differences in three vascular indices between MCDA twin and singleton groups (*p* > 0.05).

**Conclusions:**

3D-PD placental data using sonobiopsy technique could reflect the placental blood flow of each twin, which could be applied to the study of placental perfusion in MCDA twin pregnancies. This study also presented the vascular indices distribution with GA in normal twin pregnancies, which might be useful for early detection of MCDA complications.

## Background

Monochorionic diamniotic (MCDA) twin pregnancies are at higher risk of adverse outcomes than single or dichorionic diamniotic twin pregnancies, which are attributed to the influence of placental morphology in MCDA twins [1]. The complications of MCDA twin pregnancies include twin-to-twin transfusion syndrome, selective intrauterine growth restriction, twin anemia polycythemia sequence and intra-uterine demise due to uneven blood flow across placental inter-twin vascular anastomoses and/or unequal placental share [2]. Therefore, MCDA twin pregnancies need more monitoring for early detection of complications.

The placenta is an important organ to maintain pregnancy. Monitoring of placental function is an important index for clinical decisions [3]. A number of Doppler studies of placental circulation in normal and pathological pregnancies have been performed. Due to the low velocity of blood flow in the placental vascular villi, conventional color Doppler may lose some blood flow signals to reflect the placental internal perfusion. The pathological basis of abnormal blood flow in umbilical artery is the reduction of the number of placental villi. Reduction of placental blood perfusion comes earlier than the umbilical resistance index. Three-dimensional power Doppler (3D-PD) ultrasound has been used to monitor the placental perfusion, which is not affected by the angle of ultrasound detection or blood flow velocity [4]. 3D-PD, which displays vascular patterns and Doppler signals within tissues, provides estimates of three vascular indices: vascularization index (VI), flow index (FI), and vascularizationflow index (VFI) [5, 6].

3D-PD has been widely used in quantitative assessment of fetal/placental blood perfusion of singleton pregnancies [7–11], with little information in twin pregnancies due to the complexity of the placenta. In our study, we prospectively acquired the volume data of twin pregnancies using sonobiopsy technique and evaluate the vascular indices distribution with gestational age (GA), then we compared the vascular indices with those of singleton pregnancies to show the effectiveness of 3D-PD ultrasound in evaluation of placental blood flow in MCDA twin pregnancies.

## Methods

MCDA twin pregnancies (GA range, 14–32 weeks) were included in this prospective study in Shengjing hospital from August 2015 to February 2018. Chorionicity was based on first-trimester ultrasound findings and confirmed by postpartum placental pathology. GA was confirmed by last menstruation and early pregnancy ultrasound examination. Exclusion criteria were as follows: unclear display of umbilical cord insertion site; placenta previa; velamentous cord insertion; the distance between the umbilical cord insertions ≤5 cm; fetal malformations or complications; fetal abdominal circumference and/or estimated fetal weight below the tenth percentile for GA; pregnant women with obvious abnormalities or hereditary diseases. The MCDA twins that meet the criteria were included in our study. The twin pregnancies were divided into 19 subgroups according to GA; each group included 5–7 cases. Accordingly, we collected the similar number of singleton fetuses in each GA. A control group was selected from low-risk singleton pregnancies attending our hospital between gestational weeks 14 and 32 for routine ultrasound scan. After delivery, only cases with uneventful pregnancy course were considered for comparison.

This study was approved by the hospital ethics committee. All individual participants included in the study signed the informed consent. All fetuses were followed up and confirmed to be normal after birth. And the placentae of twin pregnancies were confirmed MCDA.

### Ultrasound examination

The Voluson E8 4D ultrasound system (GE Healthcare Austria GmbH & Co., Pfaffing, Austria) with a 4.0–8.0 MHz transabdominal transducer was used for sonographic examinations. An initial two-dimensional conventional study was conducted to obtain general fetal and placental information. At 3D acquisition, the following power Doppler settings were applied to standardize evaluations of placental blood flow: pulse repetition frequency, 0.9 kHz; frequency, low; quality, norm; balance, 170; filter, 2; wall motion filter, low-1; smooth, 4/5; artifact, on. Each fetal placental sample was examined in 3D rendering mode. The power Doppler sampling frame was placed on the connection point between the placenta and umbilical cord. Each gain value was set individually. The sub-noise gain setting was applied to allow the attenuation of power Doppler signal due to different maternal abdominal wall thicknesses and placental sites. At first, the maximum gain value was set and then lowered to a level that could eliminate artifacts and get an accurate image of placental blood vessels [12, 13]. In pregnancies with posterior placentae, the probe was inclined laterally to bring the placenta closer to the probe. The technique adopted a fixed 35° scanning angle to gather the 3D volume information close to each fetal umbilical cord insertions. If motion artifacts appeared, acquisition was repeated to obtain clear views of 3D-PD volumes.

**Fig. 1:**
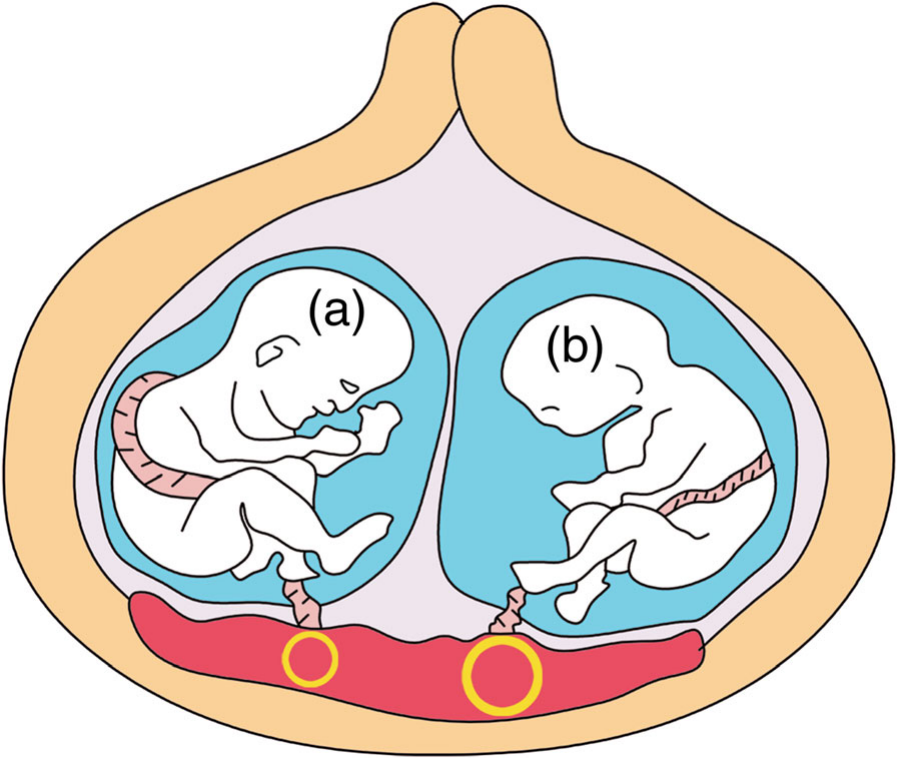
Schematic representation of three-dimensional power Doppler of each fetal placental volume in twin pregnancy. The sphere (yellow sphere frame) included placental tissue between the chorionic and basal plate close to each fetal (fetus **a** and fetus **b**) umbilical cord insertion site

**Fig. 2:**
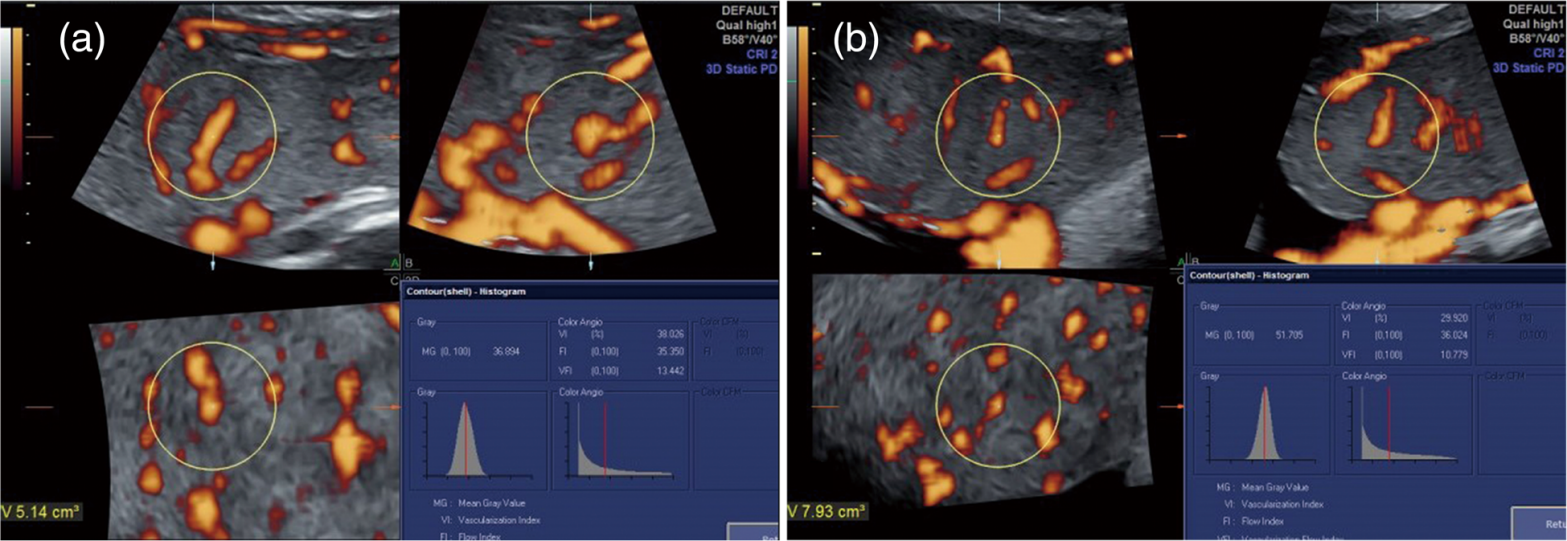
Acquisition of placental volumes with three-dimensional power Doppler in twin pregnancy. The volume data was acquired close to the umbilical cord implantation site of a normal monochorionic diamniotic twin pregnancy (fetus **a** and fetus **b**) at gestational week 32^+ 6^. The sphere volume (yellow sphere frame) varied according to placental thickness. Placental indices of fetus **a**: volume 5.14 cm^3^; VI 38.026; FI 35.35; VFI 13.442. Placental indices of fetus **b**: volume 7.93 cm^3^; VI 29.92; FI 36.024; VFI 10.779. FI, Flow index; VFI, Vascularizationflow index; VI, Vascularization index

The volumes were measured using the virtual organ computer-aided analysis (VOCAL) program (4D View; GE Healthcare Austria GmbH & Co) by one person. The technique described by Mercé et al. was used for 3D-PD evaluation of the placenta, which was a reproducible and validated method [14–16]. The obtained 3D volumes were rotated to locate the area with the highest vascular density. The sphere mode was activated to contain the placental total thickness, while excluding vessels from basal and chorionic plates (Fig. 1). Three indices (range, 0–100) were used for quantitative evaluation of placental blood perfusion of a sphere sample (Fig. 2). VI referred to the percentage of the number of color voxels in the volume of interest. FI referred to the average amplitude values of all color voxels. VFI combined the vessel presence and flow information [6]. The same 3D volume data of each twin was measured three times. The average values of VI, FI and VFI were calculated for each twin.

### Data analysis

The clinical characteristics between MCDA twin and singleton groups were compared using independent *t*-test and Chi-square testing. Difference of three vascular indices between the fetuses of each twin was compared using paired samples *t*-test. Kolmogorov–Smirnov tests were used to confirm the normal distribution of three vascular indices. The means and standard deviation values of three vascular indices per fetus were calculated and regression analysis of the vascular indices as a function of GA was performed in twin pregnancies. The vascular indices of two groups were compared using independent *t*-test. We performed a test–retest series to assess the intraobserver and interobserver variation in measurements. Thirty MCDA twins were examined in duplicate by the same examiner and re-examined by a second examiner. All examiners were blinded to all previous results. All data processing was performed with SPSS 23.0 statistical software (SPSS, Inc., Chicago, IL, USA). *P* < 0.05 was considered statistical significance.

## Results

A total of 121 MCDA twin pregnancies and 119 singleton pregnancies (GA range, 14–32 weeks) were included in the study. The volume acquisition of 17 twins and 13 singleton fetuses was unsuccessful because of fetal shadowing or frequent fetal movement. The acquisition was successful in 86% (104/121) / 89% (106/119) of twin/singleton pregnancies.

**Fig. 3:**
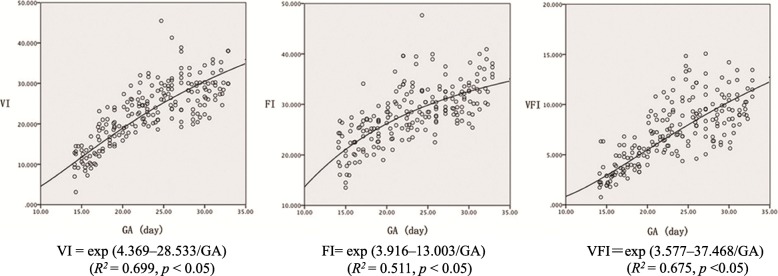
Scatterplots of vascularization index, flow index and vascularizationflow index versus gestational age. Curve regression lines indicate the mean values. VI = exp. (4.369–28.533/GA) (*R*^*2*^ = 0.699, *p* < 0.05); FI = exp. (3.916–13.003/GA) (*R*^*2*^ = 0.511, *p* < 0.05); VFI = exp. (3.577–37.468/GA) (*R*^*2*^ = 0.675, *p* < 0.05). GA, Gestational age; FI, Flow index; VFI, Vascularizationflow index; VI, Vascularization index

**Fig. 4:**
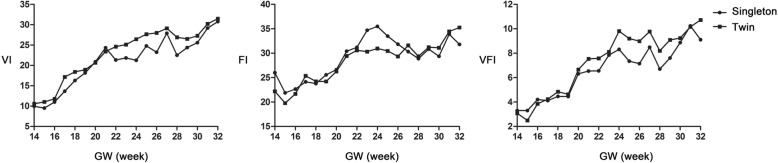
Comparisons of vascularization index, flow index and vascularizationflow index between twin and singleton pregnancies. Dots represented the mean values at each GA of singleton pregnancies. Squares represented the mean values at each GA of monochorionic diamniotic twin pregnancies. There were no significant differences of VI, FI, and VFI between MCDA twin and singleton pregnancies (*p* > 0.05). GW, Gestational week; FI, Flow index; VFI, Vascularizationflow index; VI, Vascularization index

There were no significant differences in maternal age, parity and BMI between the two groups (*p* > 0.05). Table 1 presented the comparative characteristics of subjects between the MCDA twin and singleton groups. There were no significant differences in VFI, FI, and VI between the fetuses of normal MCDA twins (*p* > 0.05). The vascular indices, VI, FI and VFI were plotted as a function of GA on a curve. The scatterplots revealed a positive relationship between the three indices and GA. The regression equations for three indices as a function of GA in days were as follows: VI = exp. (4.369–28.533/GA) (*R*^*2*^ = 0.699, *p* < 0.05), FI = exp. (3.916–13.003/GA) (*R*^*2*^ = 0.511, *p* < 0.05), and VFI = exp. (3.577–37.468/GA) (*R*^*2*^ = 0.675, *p* < 0.05) (Fig. 3). Table 2 listed the mean values and standard deviations of the three vascular indices of MCDA fetuses by GA from week 14 to 32. There were no significant differences in three vascular indices between MCDA twin and singleton groups (*p* > 0.05). Line charts displayed the comparisons of vascular values between two groups (Fig. 4).

**Table 1:** Comparative characteristic of subjects between the MCDA twin and singleton groups

	MCDA twin group	Singleton group
	(*n* = 104)	(*n* = 106)
Maternal age (years old)	29.2 ± 6.2	27.7 ± 3.4
Parity (primapara/multipara)	61 (58.7%)/43 (41.3%)	56 (53.3%)/50 (46.7%)
BMI	29.9 ± 4.3	27.6 ± 4.0
Gestational age at time of delivery (weeks)	35 ± 3.4^a^	38 ± 2.2

*BMI* body mass index, *MCDA* monochorionic diamniotic, ^a^, *p* < 0.05, data were presented compared with the singleton group

**Table 2:** Sonographic measurements of placental indices of monochorionic diamniotic twins by gestational age

GA (week)	Number of fetuses	VI			FI			VFI		
		M	-SD~ + SD	-2SD~ + 2SD	M	-SD~ + SD	-2SD~ + 2SD	M	-SD~ + SD	-2SD~ + 2SD
14~	10	10.6	7–14.2	3.4–17.8	22.2	18.5–25.9	14.8–29.6	3.1	1.2–5	−0.7-6.9
15~	12	11.1	8.9–13.3	6.7–15.5	19.8	15.2–24.4	10.6–29	2.5	2–3	1.5–3.5
16~	10	11.8	9.4–14.2	7–16.6	21.7	19.2–24.2	16.7–26.7	3.9	2.7–5.1	1.5–6.3
17~	12	17.2	13.7–20.7	10.2–24.2	25.4	21.5–29.3	17.6–33.2	4.2	2.9–5.5	1.6–6.8
18~	10	18.4	15.4–21.4	12.4–24.4	24.2	21.8–26.6	19.4–29	4.9	3.9–5.9	2.9–6.9
19~	10	18.9	15.7–22.1	12.5–25.3	24.2	20.9–27.5	17.6–30.8	4.6	3.6–5.6	2.6–6.6
20~	12	20.7	17–24.2	13.3–28.1	26.3	23.4–29.2	20.5–32.1	6.7	5.1–8.3	3.5–9.9
21~	10	23.4	19.8–27	16.2–30.6	29.4	25.3–33.5	21.2–37.6	7.5	5.9–9.1	4.3–10.7
22~	14	24.6	21–28.2	17.4–31.8	30.6	25.4–35.8	20.2–41	7.6	5.7–9.5	3.8–11.4
23~	12	25.1	20.8–29.4	16.5–33.7	30.3	25–35.6	19.7–40.9	8.1	5.6–10.6	3.1–13.1
24~	12	26.5	19.2–33.8	11.9–41.1	31	25.2–36.8	19.4–42.6	9.8	6.5–13.1	3.2–16.4
25~	10	27.7	25.9–29.5	24.1–31.3	30.5	24.8–36.2	19.1–41.9	9.2	6.8–11.6	4.4–14
26~	10	28	22.2–33.8	16.4–39.6	29.4	27–31.8	24.6–34.2	9	6.8–11.2	4.6–13.4
27~	10	29.1	22–36.2	14.9–43.3	31.6	28.5–34.7	25.4–37.8	9.8	6.8–12.8	3.8–15.8
28~	12	26.9	22.5–31.3	18.1–35.7	29.4	27.3–31.5	25.2–33.6	8.2	6.5–9.9	4.8–11.6
29~	10	26.5	22.3–30.7	18.1–34.9	31.2	28.8–33.6	26.4–36	9.1	6.9–11.3	4.7–13.5
30~	12	27.3	23.7–30.9	20.1–34.5	31.1	27.3–34.9	23.5–38.7	9.2	6.7–11.7	4.2–14.2
31~	10	30.2	26.8–33.6	23.4–37	34.4	31–37.8	27.6–41.2	10.2	8.5–11.9	6.8–13.6
32~	10	31.5	27–36	22.5–40.5	35.3	31.6–39	27.9–42.7	10.7	9.2–12.2	7.7–13.7

*FI* flow index, *GA* gestational age, *M* mean, *SD* standard deviation, *VFI* Vascularizationflow index, *VI* Vascularization index

Intra-observer variations for measurements of placental VI, FI and VFI were 5.7, 15.5 and 9.1%, respectively, and inter-observer variations were 7.9, 17.8 and 8.1%, respectively.

## Discussion

MCDA twins required more attention for early detection of complications. The placental 3D-PD indices acquired by sonobiopsy technique could reflect the placental blood flow of each twin in MCDA twin pregnancies. Our study provided reference ranges for the placental indices VI, FI, VFI in MCDA twins between gestational weeks 14 and 32, measured using 3D-PD combined with VOCAL.

Many previous reports on measurement of placental blood flow focused on singleton pregnancies. Our study showed the vascular indices increased as pregnancy progressed, consistent with some of them [14, 17]. Some scholars reported different results, due to different analytical methods. De Paula et al. analyzed the vascular indices by 3D-PD scanning the whole placental volume and presented consistent distributions throughout gestational period [16]. This method was more suitable for study of small placentae, such as early pregnancies [18, 19]. MCDA twins shared one placenta; it was difficult to distinguish the placental part of each twin. It was uneasy to scan the whole placental volume in late pregnancy with fetal shadowing. However, it was feasible to catch the site of the umbilical cord insertions of each twin. In our study, we acquired the 3D-PD placental data with Mercé’s sonobiopsy at the site of each fetal umbilical cord insertion [13]. This method could be used throughout the gestational period. There were no significant differences in VI, FI and VFI of fetuses among MCDA twin and singleton pregnancies. 3D-PD placental data using sonobiopsy technique could reflect the placental blood flow of each twin, which could be applied to the study of placental perfusion in MCDA twin pregnancies.

All MCDA placentae had inter-twin anastomoses. Three principal types of these anastomoses existed, including arteriovenous anastomoses (AVAs), arterioarterial anastomoses (AAAs) and venovenous anastomoses (VVVs). AAAs and VVVs connections were superficial without interposed villous vascular trees. During volume acquisition, we excluded the blood vessels from basal and chorionic plates. Thus, AAAs and VVVs connections had no effect on the results. AVAs ended in a shared cotyledon and existed in the vascular equator between the twins, often far from the umbilical cord insertion site. The sample volume varied according to placental thickness. In this study, placental thickness was less than 5 cm in all cases. MCDA twins with an umbilical cord entrance distance of less than 5 cm were excluded to ensure each fetal independent volume measurements.

Some reports emphasized that the application of 3D-PD should consider the impact of depth of measurement due to acoustic attenuation [20–22]. However, in some clinical studies, there were no significant differences in vascular indices based on placental position, despite the expected attenuation [6, 23, 24]. The signal loss due to attenuation by depth might be compensated by the false Doppler signal generated by the time gain control setting [25]. However, we could not calculate the amount of difference between loss of signal with attenuation and false Doppler signal. There were 18 twin pregnancies with posterior placentae, 7 twin pregnancies with fundal wall placentae in our research. These 25 twin pregnancies had a good visualization of each fetal umbilical cord insertion. We applied some methods to reduce the impact of depth. The probe was inclined laterally to bring the placenta closer to the probe. Individual adjustments of PD were applied for adequate assessment of placental blood flow [12, 13, 25]. From the third trimester on, it was hard to display the umbilical cord insertion sites of MCDA twins in fundal wall or posterior placentae. Therefore, in the third trimester, only the MCDA twins with anterior placentae were enrolled in the study.

3D-PD could display intra-placental vascular tree and quantitatively assessment of placental blood flow [14],which could not be done by 2D Doppler. The vascular indices obtained by sonobiopsy technique showed good intraclass and interclass correlation in our study. It was easy to master the 3D-PD acquisition method. Data post-analysis required time and expertise. However, time would be shortened as experience increased. Therefore, the 3D-PD technique could be applied to traditional ultrasound for monitoring placental blood perfusion in twin pregnancies.

There were some limitations to this study. It was hard to display the umbilical cord insertion sites at an older GA because of the shadowing of twins and crowed space. We didn’t get a certain amount of volume data beyond 32 weeks of gestation. Nevertheless, complications of MCDA twin pregnancies mostly occured between 15 and 26 weeks of gestation [2]. There were some limitations in the study of changes of 3D-PD placental indices in the third trimester of MCDA twin pregnancies, thus further studies with larger cohorts of women in late pregnancy were needed to confirm these results. There were 25 twin pregnancies with posterior or fundal wall placentae in our research. To some extent, the inclusion of twin pregnancies with posterior or fundal wall placentae made the result about the changes in VI, FI and VFI before GA 28 might be inaccurate due to depth attenuation.

## Conclusions

3D-PD placental data using sonobiopsy technique could reflect the placental blood flow of each twin, which could be applied to the study of placental perfusion in MCDA twin pregnancies. This study also presented the vascular indices distribution with GA in normal twin pregnancies, which might be useful for early detection of MCDA complications.

## Abbreviations

3D-PD: Three-Dimensional Power Doppler
AAAs: Arterioarterial Anastomoses
AVAs: Arteriovenous Anastomoses
FI: Flow Index
GA: Gestational Age
MCDA: Monochorionic Diamniotic
VFI: Vascularizationflow Index
VI: Vascularization Index
VOCAL: Virtual Organ Computer-Aided Analysis
VVVs: Venovenous Anastomoses
